# Many Routes to an Antibody Heavy-Chain CDR3: Necessary, Yet Insufficient, for Specific Binding

**DOI:** 10.3389/fimmu.2018.00395

**Published:** 2018-03-08

**Authors:** Sara D’Angelo, Fortunato Ferrara, Leslie Naranjo, M. Frank Erasmus, Peter Hraber, Andrew R. M. Bradbury

**Affiliations:** ^1^Specifica Inc., Santa Fe, NM, United States; ^2^Los Alamos National Laboratory, Los Alamos, NM, United States

**Keywords:** heavy-chain complementarity-determining region 3, single-chain Fv display, binding specificity, rearrangement, inverse PCR

## Abstract

Because of its great potential for diversity, the immunoglobulin heavy-chain complementarity-determining region 3 (HCDR3) is taken as an antibody molecule’s most important component in conferring binding activity and specificity. For this reason, HCDR3s have been used as unique identifiers to investigate adaptive immune responses *in vivo* and to characterize *in vitro* selection outputs where display systems were employed. Here, we show that many different HCDR3s can be identified within a target-specific antibody population after *in vitro* selection. For each identified HCDR3, a number of different antibodies bearing differences elsewhere can be found. In such *selected* populations, all antibodies with the same HCDR3 recognize the target, albeit at different affinities. In contrast, within *unselected* populations, the majority of antibodies with the same HCDR3 sequence do not bind the target. In one HCDR3 examined in depth, all target-specific antibodies were derived from the same VDJ rearrangement, while non-binding antibodies with the same HCDR3 were derived from many different V and D gene rearrangements. Careful examination of previously published *in vivo* datasets reveals that HCDR3s shared between, and within, different individuals can also originate from rearrangements of different V and D genes, with up to 26 different rearrangements yielding the same identical HCDR3 sequence. On the basis of these observations, we conclude that the same HCDR3 can be generated by many different rearrangements, but that specific target binding is an outcome of unique rearrangements and VL pairing: the HCDR3 is necessary, albeit insufficient, for specific antibody binding.

## Introduction

Antibodies bind their targets using diversified loops, termed complementarity-determining regions (CDRs), with three in each rearranged VH and VL gene. CDRs 1 and 2 are encoded by germline V genes, while CDR3s in both VH and VL are the product of gene recombination. Compared to other CDRs, the varied length and biochemical properties of heavy-chain complementarity-determining region 3 (HCDR3) contribute to enhanced sequence diversity (1). It has been estimated (2) that the theoretical HCDR3 diversity exceeds 10^15^ variants, generated from fixed genomic sequences by combinatorial and junctional diversification mechanisms. This underlies the vast diversity of the human antibody repertoire. The fully assembled V(D)J gene and its incorporated HCDR3 are derived from the sequential random assembly of 56 VH, 23 DH, and 6 JH genes (3–6). While both VH and JH contribute to the HCDR3, the DH forms the central core. Although DH genes are predominantly read in one frame, all three frames can be used (7, 8), further increasing potential diversity. It was initially thought that D genes could also be inverted and duplicated (9, 10); however, recent deep sequencing results indicate that this is unlikely (7). Diversity is further increased by P-nucleotide-mediated (11) or N-nucleotide-mediated (12, 13) addition, or exonuclease-mediated loss (11, 14), of nucleotides between the VH/DH and DH/JH segments. Recombination between VH genes after rearrangement provides further diversification although it remains unclear how much this contributes overall (15).

There is much evidence that the HCDR3 is the major determinant of antibody-binding specificity. Specific antibodies have been selected from synthetic antibody libraries where diversity is restricted to the HCDR3 (16–18). It has been shown that a greater number of antibodies were selected from a synthetic library containing only HCDR3 diversity than when the same library was combined with LCDR3 diversity (18). However, due to the random diversity in both CDR3s, this lower performance may have been due to a reduced fitness profile, caused by either the higher mutational load or potential inter-CDR structural clashes. In addition to display libraries, transgenic mice with antibody diversity restricted to the HCDR3 were able to generate high-affinity responses *in vivo* (19). HCDR3’s themselves have also been harvested as diversity elements (20–22), and low-affinity binders have been selected from fluorescent scaffold libraries in which they provide the only diversity (23). Further evidence that HCDR3s are the major determinants of antibody-binding specificity arises from the observation that peptides derived from HCDR3 structures can show biological activity similar to the antibodies from which they were derived (24, 25), in one case even demonstrating *in vivo* viral neutralization (25). Furthermore, peptide libraries generated from naïve IgM HCDR3s produce specific binders against targets (21, 26), often more efficiently than synthetic peptide libraries. HCDR3s have also been transplanted from antibodies to other proteins, conferring the expected binding activity upon those non-antibody scaffolds. These include the HCDR3 from different antibodies transplanted into neocarzinostatin (27), sfGFP (23), or an epidermal growth factor-like module of human tissue-type plasminogen activator (28). In each of these cases, the grafted HCDR3 recapitulated the antibody-binding activity. Although it is evident that the HCDR3 is critical in antigen binding, diversity confined to the LCDR3 can still generate specific antibodies (29), and it is known from affinity maturation experiments that the affinities of antibodies with identical HCDR3s may differ by up to 100-fold (30, 31).

Although the other antibody-binding loops have defined canonical structures (32–35), the prediction of the HCDR3 conformation is not trivial and has been found to have a wide variety of different possible configurations (35). In addition to the structural variability of HCDR3s with different sequences, the same HCDR3 can adopt different conformations within the same antibody when bound to different targets (36) or in uncomplexed antibodies with different VH/VL frameworks (37). This reflects the important role that HCDR3 plays in target recognition by antibodies (10, 19, 38) and likely shows that HCDR3 conformational flexibility is an additional diversity mechanism employed by the immune system.

The diverse nature of the HCDR3 has led to its use as a fingerprint both *in vivo* (39–43) and *in vitro* (44–50). In this article, we have assessed the diversity of HCDR3 sequences in an *in vitro* selected antibody population. We found that *in vitro* selection elicits hundreds of different target-specific HCDR3s, but that only within the context of a target-specific antibody population, antibodies with the same HCDR3 recognize the target. In an unselected population, we were unable to identify any sequenced antibodies with the same HCDR3 that was target specific. We conclude that the HCDR3 is necessary, but insufficient, for specific antibody binding.

## Results

### Selection of Anti-CDK2 Antibodies from a Naïve Human Recombinant Library

**Figure 1:**
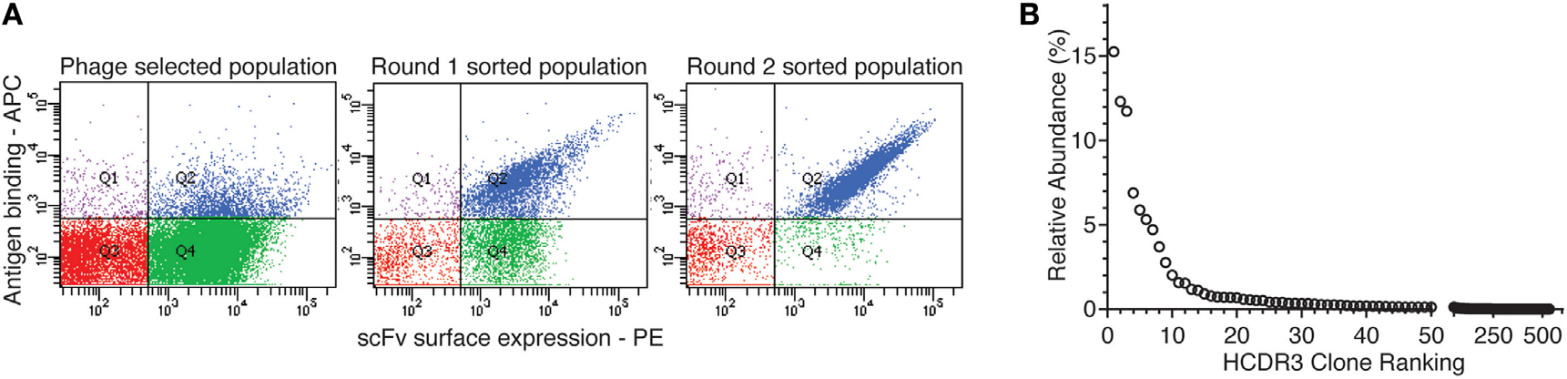
Yeast display sorting and analysis of CDK2-specific antibodies. **(A)** After two rounds of phage selection, the antibody population was displayed on yeast cells and enriched for CDK2-specific binders by two rounds of flow cytometry-assisted sorting performed at 100 nM. **(B)** Abundance distribution of the selected anti-CDK2 clones as identified by their heavy-chain complementarity-determining region 3 (HCDR3s) by next-generation sequencing.

We selected antibodies against CDK2, a human cyclin-dependent kinase, provided by the Structural Genomic Consortium (SGC; Toronto), from a well-validated (44, 45, 51–59) large naïve phage antibody library in the single-chain Fv (scFv) format, created by site-specific recombination (59, 60). This library was previously used to develop a combined phage and yeast display approach (45, 53), which has the advantage that many more antibodies can be identified than by regular phage display, particularly when combined with next-generation sequencing (NGS) (44). After two rounds of phage selection (using biotinylated CDK2 antigen and streptavidin-coated magnetic beads) and two rounds of yeast sorting (at 100 nM antigen concentration), almost all yeast displaying antibodies recognized the target, as shown in Figure 1A.

The HCDR3s of the final sorted population were sequenced using IonTorrent. 32,138 total HCDR3 sequences were obtained and analyzed with the Antibody Mining Toolbox (61). 535 different HCDR3s aa sequences made up 98% of all the sequences analyzed. The remaining 2% of sequences mainly comprised HCDR3 represented by one or two sequences and were ignored for the purposes of this study as the result of possible sequencing errors. The 535 HCDR3 sequences were ranked by abundance, and their distribution is presented in Figure 1B. The majority of HCDRs were represented by limited numbers of clones.

In a previous publication (44), we described the identification and affinity determination of representative clones of 8 of the 10 most abundant HCDR3 clones. All isolated clones bound CDK2, and for each HCDR3 sequence identified, the affinity of one representative clone was assessed directly on the yeast surface (62). The affinities of these eight most abundant identified clones were found to range from 2 to 75 nM, as previously described (44).

### HCDR3-Based Rescue of Anti-CDK2 Antibodies and Characterization

To assess whether an identified HCDR3 corresponded to a single clone, or a sublibrary of clones, we used an inverse PCR approach anchored within the most abundant unique HCDR3 sequence (50) to isolate all clones containing this HCDR3 within the sorting-enriched population.

**Figure 2:**
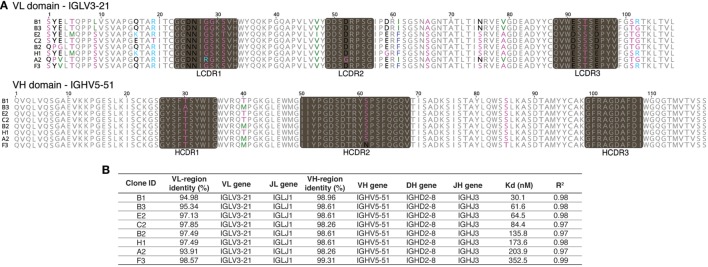
Sequences and affinities of single-chain Fvs with an identical heavy-chain complementarity-determining region 3 (HCDR3). Inverse PCR of the most abundant clone of the selected population was analyzed by Sanger sequencing. **(A)** Sanger sequence analysis of the eight unique clones with identical HCDR3 obtained by inverse PCR. **(B)** VDJ gene usage and affinity values of the eight different clones.

When 24 random clones from this sublibrary were Sanger sequenced, eight different antibodies, all containing the same HCDR3, were identified. Additional mutations were identified in the rest of the VH, and significantly more in the VL. In fact, for the VL, four different CDR1, two different CDR2 and two different CDR3 sequences, were identified (Figure 2A). However, the different clones shared 91.6–97.8% homology, and the same rearrangements were responsible for VH (5-51, D2-08, and J3) and VL (IGLV3-21 and IGLJ1). Finally, when the affinities of the eight expressing and binding clones were calculated, they spanned a 10-fold range (from 30.1 to 352.5 nM) (Figure 2B), reflecting the 100 nM target concentration used for sorting.

### HCDR3-Based Rescue of Non-Selected Antibodies and Characterization

**Figure 3:**
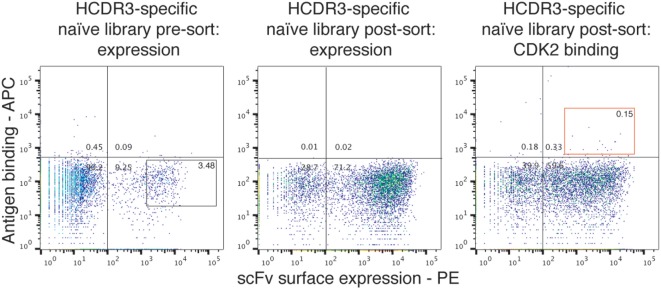
Analysis of the naïve library for single-chain Fvs (scFvs) with identical heavy-chain complementarity-determining region 3 (HCDR3s). Yeast clones displaying scFvs from the HCDR3-specific mini-library obtained from the naïve library *via* inverse PCR were analyzed by flow cytometry. Cells expressing scFvs on the surface and detected by anti-SV5-PE antibody were gated and sorted (left panel). Once grown and analyzed, they showed an increase in the expressing population (middle panel) with a limited, but detectable, binding for biotinylated CDK2 detected by streptavidin-APC conjugation (right panel).

**Figure 4:**
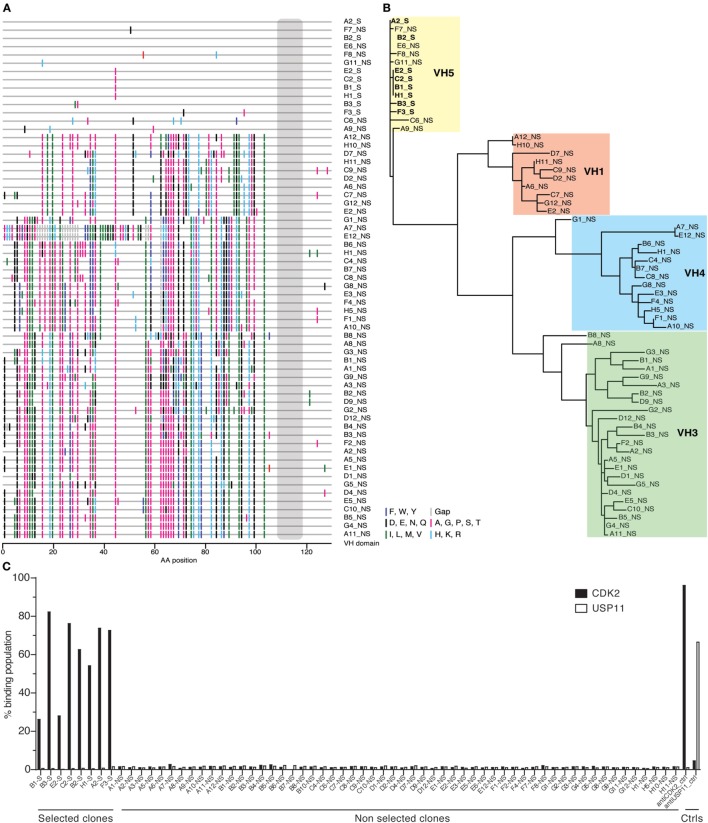
Analysis of antibody sequences obtained by inverse PCR. **(A)** The alignment of antibody sequences with the same heavy-chain complementarity-determining region 3 (HCDR3) from naïve and selected libraries shows differences in VH gene sequences. This representation, a so-called highlighter plot, indicates amino acid mutations in the sites that differ from the first sequence. The alignment was generated with the Highlighter Tool (hiv.lanl.gov/content/sequence/HIGHLIGHT/highlighter_top.html). **(B)** The sequences of the antibodies sharing the same HCDR3 obtained by inverse PCR from the population enriched for specific antigen binders were phylogenetically clustered to show relations among variants that share VH alleles. **(C)** Single-chain Fvs (scFvs) sharing the same HCDR3 but obtained from either the selected or the naïve population were displayed on yeast and their binding activity was measured by flow cytometry and reported in the histogram as percentage of binding. None of the scFvs isolated from the naïve library bound CDK2, as opposed to the ones from the selected population.

Given this broad variation in affinity, and the known importance of the HCDR3 in antibody binding specificity, we applied the inverse PCR technique to the original naïve, unselected library to assess the relative abundance of clones containing the HCDR3 sequence of interest. The inverse PCR reaction, using primers specific for the top-ranked clone, was performed using a plasmid preparation of the naïve library as a template. The obtained mini-library was transformed into yeast-competent cells, and, upon induction, the cells were sorted for well-displayed antibodies (Figure 3, left and middle panels). Of note, after sorting for expression and analyzing such population for binding to CDK2 (Figure 3, third panel), only 0.15% of the clones showed binding for the cognate antigen. Ninety six clones of the population sorted for expression were sequenced. We identified 55 different scFvs containing the same HCDR3 with a far greater variation in both VH and VL than seen in the clones isolated from the selected population: 37.7–73.2% homology to the selected clones. Figure 4A shows the alignment of the VH regions of the sequenced clones. The VL families, not being under any particular selective pressure, were very diverse, derived from 12 VL-kappa and 10 VL-lambda germline genes, with 4 JL-kappa genes and 3 JL-lambda genes represented. The VH genes, on the other hand, having all been selected to contain the same HCDR3 sequence, were, not surprisingly, all found to have the same JH and DH genes (with one exception). More surprising was the diversity of the VH germline genes, which comprised 5 VH families derived from 19 different germline VH genes (Figure 4B; Table 1).

**Table 1:** Analyses of the gene usage from the heavy-chain complementarity-determining region 3 (HCDR3)-specific non-selected population.

Clone ID	VH gene	DH gene	JH gene	HCDR3	VL gene	JL gene	LCDR3
C7	IGHV1-2	IGHD2-8	IGHJ3	CAKGFRAGDAFDIW	IGKV2-28	IGKJ3	CMQTLQTPFTF
G12	IGHV1-2	IGHD2-8	IGHJ3	CAKGFRAGDAFDIW	IGLV2-14	IGLJ1	CSSYTSVSTYVF
E2	IGHV1-2	IGHD2-8	IGHJ3	CAKGFRAGDAFDIW	IGLV7-46	IGLJ2	CLLDYTDARVF
D7	IGHV1-3	IGHD2-8	IGHJ3	CAKGFRAGDAFDIW	IGKV1-39	IGKJ1	CQQSYSTPWTF
A6	IGHV1-46	IGHD2-8	IGHJ3	CAKGFRAGDAFDIW	IGKV2-28	IGKJ4	CMQSLQTPLTF
C9	IGHV1-46	IGHD2-8	IGHJ3	CAKGFRAGDAFDIW	IGLV2-8	IGLJ2	CSSYAGSNNVVF
D2	IGHV1-46	IGHD2-8	IGHJ3	CAKGFRAGDAFDIW	IGLV2-14	IGLJ1	CCSYGGPYVF
H11	IGHV1-46	IGHD2-8	IGHJ3	CAKGFRAGDAFDIW	IGKV1-5	IGKJ4	CQQYYSTPLTF
A12	IGHV1-69	IGHD2-8	IGHJ3	CAKGFRAGDAFDIW	IGLV1-40	IGLJ1	CQSYDSSLSGYVF
H10	IGHV1-69	IGHD2-8	IGHJ3	CAKGFRAGDAFDIW	IGLV2-18	IGLJ1	CSSYTSSSTYVF
B2	IGHV3-21	IGHD2-8	IGHJ3	CAKGFRAGDAFDIW	IGKV1-12	IGKJ3	CQQTNSFPFTF
D9	IGHV3-21	IGHD2-8	IGHJ3	CAKGFRAGDAFDIW	IGKV1-9	IGKJ5	CHQTDTLPITF
A11	IGHV3-23	IGHD2-8	IGHJ3	CAKGFRAGDAFDIW	IGKV1-5	IGKJ2	CQQYDTLPRTF
B5	IGHV3-23	IGHD2-8	IGHJ3	CAKGFRAGDAFDIW	IGKV1-39	IGKJ4	CQQSYSTPPTF
G4	IGHV3-23	IGHD2-8	IGHJ3	CAKGFRAGDAFDIW	IGKV4-1	IGKJ2	CQQYHSTPYTF
A2	IGHV3-23	IGHD2-8	IGHJ3	CAKGFRAGDAFDIW	IGKV1-5	IGKJ2	CQQYVECSF
A5	IGHV3-23	IGHD2-8	IGHJ3	CAKGFRAGDAFDIW	IGKV2-28	IGKJ2	CMQALQSPRTF
A8	IGHV3-23	IGHD2-8	IGHJ3	CAKGFRAGDAFDIW	IGKV3-11	IGKJ2	CQQYNNWPPYTF
B4	IGHV3-23	IGHD2-8	IGHJ3	CAKGFRAGDAFDIW	IGKV1-17	IGKJ4	CLQHNLYPRTF
D1	IGHV3-23	IGHD2-8	IGHJ3	CAKGFRAGDAFDIW	IGKV3D-15	IGKJ4	CQQYNNWPPLTF
D4	IGHV3-23	IGHD2-8	IGHJ3	CAKGFRAGDAFDIW	IGKV3D-20	IGKJ2	CQQFGGSPKCSF
E1	IGHV3-23	IGHD2-8	IGHJ3	CAKGFRAGDAFDIW	IGKV1-17	IGKJ4	CLQHNTYPLTF
F2	IGHV3-23	IGHD2-8	IGHJ3	CAKGFRAGDAFDIW	IGKV2-28	IGKJ1	CMQALQTPWTF
G2	IGHV3-23	IGHD2-8	IGHJ3	CAKGFRAGDAFDIW	IGLV3-1	IGLJ2	CQVWDSNSHVVF
G5	IGHV3-23	IGHD2-8	IGHJ3	CAKGFRAGDAFDIW	IGKV3D-20	IGKJ5	CQQRSNWPLTF
B3	IGHV3-23	IGHD2-8	IGHJ3	CAKGFRAGDAFDIW	IGLV2-14	IGLJ2	CAAWDSSLSAVVF
A1	IGHV3-30	IGHD2-8	IGHJ3	CAKGFRAGDAFDIW	IGKV1-33	IGKJ5	CQQYDKLPLTF
B1	IGHV3-30	IGHD2-8	IGHJ3	CAKGFRAGDAFDIW	IGKV1-12	IGKJ2	CQQGYSFPRTF
D12	IGHV3-53	IGHD2-8	IGHJ3	CAKGFRAGDAFDIW	IGLV3-21	IGLJ2	CQAWDTHDDPWGVF
E5	IGHV3-64	IGHD2-8	IGHJ3	CAKGFRAGDAFDIW	IGKV1-33	IGKJ2	CVQHRGYPRYTF
C10	IGHV3-64D	IGHD2-8	IGHJ3	CAKGFRAGDAFDIW	IGKV3-11	IGKJ3	CQQRINRVTF
A3	IGHV3-7	IGHD2-8	IGHJ3	CAKGFRAGDAFDIW	IGKV3D-20	IGKJ3	CQQYSLYPLSF
G9	IGHV3-7	IGHD2-8	IGHJ3	CAKGFRAGDAFDIW	IGLV1-44	IGLJ1	CQAWDSRTAVF
B8	IGHV3-72	IGHD2-8	IGHJ3	CAKGFRAGDAFDIW	IGKV1-9	IGKJ4	CQQLNSYPLAF
G3	IGHV3-9	IGHD2-8	IGHJ3	CAKGFRAGDAFDIW	IGKV3-11	IGKJ5	CQQRGNWPPGATF
G1	IGHV4-31	IGHD2-8	IGHJ3	CAKGFRAGDAFDIW	IGLV3-1	IGLJ2	CQAWDSGTVVF
B6	IGHV4-31	IGHD2-8	IGHJ3	CAKGFRAGDAFDIW	IGKV2-28	IGKJ2	CMQALQSPRTF
H1	IGHV4-31	IGHD2-8	IGHJ3	CAKGFRAGDAFDIW	IGLV3-1	IGLJ2	CQAWDSSTAVF
F1	IGHV4-34	IGHD2-8	IGHJ3	CAKGFRAGDAFDIW	IGKV1-17	IGKJ1	CLQHNNYPRTF
G8	IGHV4-34	IGHD2-8	IGHJ3	CAKGFRAGDAFDIW	IGKV2D-29	IGKJ2	CMQGTHWPRTF
H5	IGHV4-34	IGHD2-8	IGHJ3	CAKGFRAGDAFDIW	IGKV4-1	IGKJ4	CQQYYSTPLTF
A10	IGHV4-34	IGHD2-8	IGHJ3	CAKGFRAGDAFDIW	IGKV1-17	IGKJ1	CLQHNNYPRTF
E3	IGHV4-34	IGHD2-8	IGHJ3	CAKGFRAGDAFDIW	IGKV2-28	IGKJ1	CMQALQAPWTL
F4	IGHV4-34	IGHD2-8	IGHJ3	CAKGFRAGDAFDIW	IGLV3-21	IGLJ2	CQVWDSRDQHVAF
A7	IGHV4-34	IGHD2-8	IGHJ3	CAKGFRAGDAFDIW	IGKV1-33	IGKJ2	CQQYDNLRYSF
E12	IGHV4-34	IGHD2-8	IGHJ3	CAKGFRAGDAFDIW	IGKV3-11	IGKJ2	CQQRSNSPPFTF
C4	IGHV4-4	IGHD2-8	IGHJ3	CAKGFRAGDAFDIW	IGLV1-51	IGLJ3	CGTWDSSLSAGVF
B7	IGHV4-59	IGHD3-10	IGHJ3	CAKGFRAGDAFDIW	IGKV4-1	IGKJ3	CQQFYSTPPLFTF
C8	IGHV4-61	IGHD2-8	IGHJ3	CAKGFRAGDAFDIW	IGLV2-8	IGLJ2	CSSYTGSNNWRVVF
A9	IGHV5-51	IGHD2-8	IGHJ3	CAKGFRAGDAFDIW	IGKV1-5	IGKJ3	CQQSYSTPLFTF
C6	IGHV5-51	IGHD2-8	IGHJ3	CAKGFRAGDAFDIW	IGKV1-5	IGKJ4	CLQHDEYPLTF
E6	IGHV5-51	IGHD2-8	IGHJ3	CAKGFRAGDAFDIW	IGKV1-5	IGKJ4	CQQADSVPLTF
F7	IGHV5-51	IGHD2-8	IGHJ3	CAKGFRAGDAFDIW	IGKV4-1	IGKJ3	CQQYSSIPFTF
F8	IGHV5-51	IGHD2-8	IGHJ3	CAKGFRAGDAFDIW	IGLV7-43	IGLJ3	CLLYYGGAQLGVF
G11	IGHV5-51	IGHD2-8	IGHJ3	CAKGFRAGDAFDIW	IGKV1-39	IGKJ1	SQQSYDSPPMTF

Testing of the scFvs by flow cytometry from the selected and naïve populations revealed that none of the scFvs containing this HCDR3 isolated from the naïve library were able to bind CDK2, while all those from the selected population bound CDK2 (Figure 4C).

We were surprised that the same HCDR3 could be assembled from so many different germline VH genes in the naïve unselected library. As the library we used was originally created by cloning the rearranged VH and VL genes of peripheral blood lymphocytes from 40 donors, this convergent HCDR3 assembly may be a normal consequence of the generation of antibody diversity, or it could be a result of the various PCR reactions we performed to create the library, as well as the final inverse PCR anchored within the HCDR3.

### *In Vivo* HCDR3 Generation

In order to assess the prevalence of identical HCDR3s derived from different germline genes *in vivo*, we analyzed two publicly available datasets of naïve B cell sequences (63, 64), referred to respectively as the “DeKosky” and the “DeWitt” datasets. In the first, ~55,000 naïve VH sequences from three donors were obtained by paired end MiSeq reads, and 23 HCDR3 sequence pairs were found to be shared between two of the three donors in the naïve repertoires. This represents a frequency of 0.083% shared HCDR3s. No HCDR3 was found to be shared among all three donors. Interestingly, all of these 23 HCDR3 pairs were discordant for identified VH germline genes, and seven were also discordant for the identified DH gene (Table 2A). However, all pairs share the same JH gene. In the second analysis (64), based on a dataset of 8,596,145 productive MiSeq reads comprising 7,984,053 unique HCDR3s from the naïve B cells of three donors, we identified 568 identical HCDR3s (0.007% of the total unique HCDR3s) generated by different VDJ recombinations (as determined by IMGT). These were generated using from two to 26 different VDJ combinations (see Table S1 in Supplementary Material), and 176 of these rearrangements were found in all three donors. Two of the HCDR3s with the greatest number of rearrangements are illustrated in Table 2B. The first, CARDRGDYW, was generated from 14 different VH genes (from five different VH gene families), five different DH genes and one JH gene in 26 different combinations. Five of these combinations were found in two donors, and two were found in all three donors, the remaining were unique combinations found in individual donors. The second, CARDSSGWYYFDYW, was a longer HCDR3 and was generated from 20 different VH genes (from all seven different VH gene families) and only one DH and one JH gene. Six of the combinations were found in two donors and four in all three donors.

**Table 2:** Analyses of (A) DeKosky and (B) DeWitt data for identical HCDR3s.

**(A)**						

**HCDR3 aa sequence**	**Length**	**VH family**	**VH gene**	**DH gene**		**JH gene**

CAKDGYW	5	IGHV01	IGHV1-46			IGHJ4
CAKDGYW	5	IGHV03	IGHV3-23			IGHJ4
CARADDAFDIW	9	IGHV01	IGHV1-69			IGHJ3
CARADDAFDIW	9	IGHV04	IGHV4-34	IGHD1-1		IGHJ3
CARALYYFDYW	9	IGHV01	IGHV1-46	IGHD1-7		IGHJ4
CARALYYFDYW	9	IGHV01	IGHV1-2	IGHD3-16		IGHJ4
CARDKYYFDYW	9	IGHV01	IGHV1-69	IGHD1-14		IGHJ4
CARDKYYFDYW	9	IGHV04	IGHV4-59			IGHJ4
CARDLDYW	6	IGHV03	IGHV3-11			IGHJ4
CARDLDYW	6	IGHV03	IGHV3-33			IGHJ4
CARDPFDYW	7	IGHV01	IGHV1-69			IGHJ4
CARDPFDYW	7	IGHV01	IGHV1-46			IGHJ4
CARDPGPW	6	IGHV01	IGHV1-69	IGHD1-14		IGHJ5
CARDPGPW	6	IGHV03	IGHV3-33	IGHD1-14		IGHJ5
CARDRSSSFDYW	10	IGHV03	IGHV3-33	IGHD6-13		IGHJ4
CARDRSSSFDYW	10	IGHV03	IGHV3-74	IGHD6-13		IGHJ4
CARDSGNDYW	8	IGHV07	IGHV7-4	IGHD6-13		IGHJ4
CARDSGNDYW	8	IGHV01	IGHV1-8	IGHD4-23		IGHJ4
CARDSSGYFDYW	10	IGHV01	IGHV1-46	IGHD3-22		IGHJ4
CARDSSGYFDYW	10	IGHV01	IGHV1-18	IGHD3-22		IGHJ4
CARDYCSGGSCYFDYW	14	IGHV04	IGHV4-31	IGHD2-15		IGHJ4
CARDYCSGGSCYFDYW	14	IGHV03	IGHV3-21	IGHD2-15		IGHJ4
CARGAAPDYW	8	IGHV01	IGHV1-46	IGHD5-12		IGHJ4
CARGAAPDYW	8	IGHV03	IGHV3-53	IGHD2-15		IGHJ4
CARGAYYFDYW	9	IGHV04	IGHV4-59	IGHD3-16		IGHJ4
CARGAYYFDYW	9	IGHV03	IGHV3-33			IGHJ4
CARGGNWFDPW	9	IGHV04	IGHV4-34	IGHD3-10		IGHJ5
CARGGNWFDPW	9	IGHV04	IGHV4-30	IGHD2-15		IGHJ5
CARGGYGDYVDYW	11	IGHV01	IGHV1-46	IGHD4-17		IGHJ4
CARGGYGDYVDYW	11	IGHV01	IGHV1-18	IGHD4-17		IGHJ4
CARGIAAADYW	9	IGHV03	IGHV3-48	IGHD6-13		IGHJ4
CARGIAAADYW	9	IGHV01	IGHV1-69	IGHD6-13		IGHJ4
CARGRVFDYW	8	IGHV04	IGHV4-34	IGHD3-16		IGHJ4
CARGRVFDYW	8	IGHV02	IGHV2-5	IGHD1-26		IGHJ4
CARGSSFDYW	8	IGHV04	IGHV4-59	IGHD3-10		IGHJ4
CARGSSFDYW	8	IGHV03	IGHV3-53	IGHD6-6		IGHJ4
CARGVAARDYW	9	IGHV04	IGHV4-59	IGHD6-6		IGHJ4
CARGVAARDYW	9	IGHV03	IGHV3-48	IGHD6-6		IGHJ4
CARRFDPW	6	IGHV03	IGHV3-21			IGHJ5
CARRFDPW	6	IGHV04	IGHV4-34			IGHJ5
CARRLGNWYFDLW	11	IGHV03	IGHV3-11	IGHD3-10		IGHJ2
CARRLGNWYFDLW	11	IGHV04	IGHV4-61	IGHD7-27		IGHJ2
CARVGSGWYFDYW	11	IGHV03	IGHV3-66	IGHD6-19		IGHJ4
CARVGSGWYFDYW	11	IGHV03	IGHV3-7	IGHD6-19		IGHJ4
CASNDAFDIW	8	IGHV01	IGHV1-46			IGHJ3
CASNDAFDIW	8	IGHV05	IGHV5-10			IGHJ3

**(B)**						

**HCDR3 aa Sequence**	**# rearrangements**	**VH family**	**VH gene**	**DH gene**	**JH gene**	**Donor representation**

CARDRGDYW	26	IGHV01	IGHV01-02	IGHD03-10	IGHJ04-01	donor1
CARDRGDYW	26	IGHV01	IGHV01-02	IGHD05-24	IGHJ04-01	donor3
CARDRGDYW	26	IGHV01	IGHV01-03	IGHD01-26	IGHJ04-01	donor3
CARDRGDYW	26	IGHV01	IGHV01-03	IGHD03-10	IGHJ04-01	donor3
CARDRGDYW	26	IGHV01	IGHV01-03	IGHD06-25	IGHJ04-01	donor1
CARDRGDYW	26	IGHV01	IGHV01-18	IGHD03-10	IGHJ04-01	donor3
CARDRGDYW	26	IGHV01	IGHV01-18	IGHD03-16	IGHJ04-01	donor1, donor3
CARDRGDYW	26	IGHV01	IGHV01-18	IGHD05-24	IGHJ04-01	donor2
CARDRGDYW	26	IGHV01	IGHV01-18	IGHD06-25	IGHJ04-01	donor3
CARDRGDYW	26	IGHV01	IGHV01-46	IGHD03-10	IGHJ04-01	donor1, donor2, donor3
CARDRGDYW	26	IGHV01	IGHV01-46	IGHD03-16	IGHJ04-01	donor2
CARDRGDYW	26	IGHV01	IGHV01-69	IGHD03-10	IGHJ04-01	donor1, donor2, donor3
CARDRGDYW	26	IGHV01	IGHV01-69	IGHD03-16	IGHJ04-01	donor2
CARDRGDYW	26	IGHV03	IGHV03-11	IGHD03-10	IGHJ04-01	donor1
CARDRGDYW	26	IGHV03	IGHV03-11	IGHD03-16	IGHJ04-01	donor1
CARDRGDYW	26	IGHV03	IGHV03-13	IGHD03-10	IGHJ04-01	donor1
CARDRGDYW	26	IGHV03	IGHV03-48	IGHD03-10	IGHJ04-01	donor3
CARDRGDYW	26	IGHV03	IGHV03-53	IGHD03-10	IGHJ04-01	donor1, donor3
CARDRGDYW	26	IGHV03	IGHV03-53	IGHD03-16	IGHJ04-01	donor3
CARDRGDYW	26	IGHV03	IGHV03-53	IGHD05-24	IGHJ04-01	donor1
CARDRGDYW	26	IGHV03	IGHV03-64	IGHD03-10	IGHJ04-01	donor1, donor3
CARDRGDYW	26	IGHV03	IGHV03-66	IGHD03-10	IGHJ04-01	donor2, donor3
CARDRGDYW	26	IGHV04	IGHV04-39	IGHD03-10	IGHJ04-01	donor1, donor3
CARDRGDYW	26	IGHV04	IGHV04-39	IGHD03-16	IGHJ04-01	donor3
CARDRGDYW	26	IGHV05	IGHV05-51	IGHD03-16	IGHJ04-01	donor2
CARDRGDYW	26	IGHV07	IGHV07-04	IGHD03-10	IGHJ04-01	donor3
CARDSSGWYYFDYW	20	IGHV01	IGHV01-02	IGHD06-19	IGHJ04-01	donor1, donor2, donor3
CARDSSGWYYFDYW	20	IGHV01	IGHV01-03	IGHD06-19	IGHJ04-01	donor1, donor2, donor3
CARDSSGWYYFDYW	20	IGHV01	IGHV01-08	IGHD06-19	IGHJ04-01	donor1
CARDSSGWYYFDYW	20	IGHV01	IGHV01-18	IGHD06-19	IGHJ04-01	donor1, donor2, donor3
CARDSSGWYYFDYW	20	IGHV01	IGHV01-46	IGHD06-19	IGHJ04-01	donor1, donor3
CARDSSGWYYFDYW	20	IGHV01	IGHV01-69	IGHD06-19	IGHJ04-01	donor2, donor3
CARDSSGWYYFDYW	20	IGHV02	IGHV02-70	IGHD06-19	IGHJ04-01	donor1, donor3
CARDSSGWYYFDYW	20	IGHV03	IGHV03-11	IGHD06-19	IGHJ04-01	donor1
CARDSSGWYYFDYW	20	IGHV03	IGHV03-20	IGHD06-19	IGHJ04-01	donor1
CARDSSGWYYFDYW	20	IGHV03	IGHV03-23	IGHD06-19	IGHJ04-01	donor3
CARDSSGWYYFDYW	20	IGHV03	IGHV03-48	IGHD06-19	IGHJ04-01	donor3
CARDSSGWYYFDYW	20	IGHV03	IGHV03-53	IGHD06-19	IGHJ04-01	donor1, donor2, donor3
CARDSSGWYYFDYW	20	IGHV03	IGHV03-64	IGHD06-19	IGHJ04-01	donor3
CARDSSGWYYFDYW	20	IGHV03	IGHV03-66	IGHD06-19	IGHJ04-01	donor2, donor3
CARDSSGWYYFDYW	20	IGHV03	IGHV03-72	IGHD06-19	IGHJ04-01	donor3
CARDSSGWYYFDYW	20	IGHV03	IGHV03-74	IGHD06-19	IGHJ04-01	donor1, donor2
CARDSSGWYYFDYW	20	IGHV04	IGHV04-39	IGHD06-19	IGHJ04-01	donor3
CARDSSGWYYFDYW	20	IGHV05	IGHV05-51	IGHD06-19	IGHJ04-01	donor3
CARDSSGWYYFDYW	20	IGHV06	IGHV06-01	IGHD06-19	IGHJ04-01	donor1, donor2
CARDSSGWYYFDYW	20	IGHV07	IGHV07-04	IGHD06-19	IGHJ04-01	donor3

## Discussion

Next-generation sequencing has been widely applied to many areas of human immunology, helping, for instance, to increase understanding of antibody repertoires (64–71), VH/VL pairing (39, 67), humoral responses to pathogens (72–74), vaccination (41, 73, 75, 76), and the role of antibodies in autoimmune conditions (77) and cancer (78). In addition to its role in understanding natural *in vivo* humoral responses, NGS has also been used in the practice of *in vitro* antibody selection, including in the sequencing of antibodies selected by phage (47–49) and yeast (44, 50) display, as well as in the analysis of naïve antibody libraries (61, 79, 80). We have previously shown (61) that the number of specific antibody HCDR3s that can be identified using NGS after a combined phage/yeast selection protocol far exceeds the number that can be isolated using standard low-throughput analysis and sequencing methods. After *in vitro* selections, we routinely use the HCDR3s as unique identifiers to rank antibody abundance. Identified clones can then be isolated by inverse PCR (50).

In the work presented here, we show that 535 different HCDR3s are identified by NGS of a yeast displayed population that is positive for binding to CDK2 (Figure 1A). This mirrors previous work in which we showed that hundreds of different HCDR3s were able to mediate specific binding against a number of different targets (44). When antibodies containing the most abundant HCDR3 were isolated from the *selected* pool using specific inverse primers, a single scFV gene was not obtained, but an “oligoclonal” population of specific binders, comprising at least eight different antibody sequences. These are all very similar to one another (91.6–97.8% homology), with most of the variation occurring in the VL but with the germline VH, DH, JH, VL, and JL genes identified as being identical. Analysis of these clones revealed a 10-fold difference in affinity (Kd), confirming the importance of additional antibody structure beyond the HCDR3 in modulating binding activity (30, 31) and indicating that the true diversity of anti-CDK2 antibodies could be significantly higher than 535, when variability in VL and HCDR1 and HCDR2 is also taken into account.

Given the identification of different antibodies with identical HCDR3s, all of which bound the target in the selected population, we turned to the naïve library to assess whether the same HCDR3 within the context of different antibodies would also be able to bind the target. By using the same inverse PCR approach, a far more diverse collection of antibodies, all of which contained the same HCDR3, was isolated. However, none of these were able to bind the target, and analysis of the aligned sequences revealed that apart from the identical HCDR3’s, these antibodies comprised very different VL genes. This was not surprising since VLs were randomly recombined and not under selective pressure. More surprising was the finding that the frameworks and CDR1 and CDR2 of the VHs were largely diverse, corresponding to 19 different germline VH genes. When this population was tested for binding to CDK2 by flow cytometry, only 0.15% of displayed antibodies with the identical HCDR3 bound the target. On the basis of these findings, we conclude that a specific HCDR3 will only define a particular binding specificity within a very narrow structurally appropriate context: i.e., HCDR3 is necessary, but is insufficient to define specific antibody-binding properties unless combined with appropriate VL and VH germline genes. This is perhaps not surprising given a recent report in which structural analysis of the same HCDR3 sequence placed within the context of different VH and VL genes shows significant conformational diversity (37). Those results, along with those presented here, suggest that the conformations of HCDR3 conformations are modified not only by their sequences but also by the structural environment in which they are found: in particular their VH and VL pairing.

It is remarkable that so many different rearranged VH genes, derived from 19 germline genes, were found to contain the same HCDR3. This begs the question as to whether the generation of identical HCDR3s from different germline genes is biological in nature, or a result of the molecular biological manipulations we had undertaken in these experiments. In a couple of published NGS analyses of *in vivo* naïve B cell HCDR3 repertoires (63, 64), 0.04–0.08% of HCDR3s were found to be shared between any pairs of donors. Further analysis of the sequences described in the study by DeKosky et al. (63) (Table 2A) reveals that *all* these so-called public HCDR3s were derived from different germline VH (and in some cases DH) genes, suggesting that the generation of identical HCDR3 sequences is stochastic and usually occurs using different germline VH and DH combinations. This conclusion was confirmed and extended by a much larger second dataset (64), which was generated by sequencing the naïve B cell repertoire of three individuals at far greater depth (>8.5 M productive reads total). Different rearrangements encoding identical HCDR3s were found both within and between donors. Altogether, 568 different HCDR3s generated with from 2 to 26 different rearrangements were identified (see Table S1 in Supplementary Material). Of these, 176 rearrangements, comprising 155 different HCDR3s, were found in all 3 donors. In a particularly notable example, the same four rearrangements (using four different VH genes and the same DH and JH genes) were found in all three donors for two of these HCDR3s (CARGYSSGWYYFDYW and CARDSSGWYYFDYW) (see Table S1 in Supplementary Material; Table 2B). These results demonstrate that the creation of identical HCDR3s from different VH or DH germline genes is a regular, albeit rare, occurrence *in vivo* and that the sequences of the HCDR3s, as well as the rearrangements used to create them, are shared among different individuals. The observed *in vitro* HCDR3 rearrangement diversity, therefore, more likely reflects the original *in vivo* recombination, rather than the consequence of molecular biological manipulation. This is further confirmed by *in vitro* selection experiments from natural naïve libraries (81), in which it was found that antibodies with the same HCDR3 sequence were derived using different VH genes.

The library used here (59) was created from the rearranged V genes of 40 donors and is estimated to comprise approximately 3.3 × 10^6^ different HCDR3s (61). The *in vivo* data described above suggest that most, if not all, of the identical HCDR3s identified in the naïve library were stochastically derived from different germline VH, DH, and JH gene rearrangements in the original donors. However, it cannot be excluded that this natural diversity was supplemented by some of the *in vitro* molecular biological manipulations we have carried out. In particular, the inverse PCR primers we used to isolate all identical HCDR3s may be “correcting” slightly different sequences to the desired HCDR3, even if, given the primer lengths (18–23 bases), they would have to be extremely similar to be able to do this. Furthermore, inadvertent PCR errors may have increased the apparent diversity of the surrounding VH gene despite the use of a proofreading polymerase. One surprising result was the low percentage (0.15%) of CDK2-binding clones containing the identified HCDR3 in the naïve library.

The earliest naïve *in vitro* antibody libraries (16, 82, 83) had claimed diversities of ~5 × 10^7^, and an average of ~4 antibodies were selected per target. A smaller subset (10^7^) of a much larger library yielded ~1 antibody per target (84). Assuming the diversity estimates for the sizes of these (sub)libraries is correct, these results suggest that one should expect one positive antibody per ~10^7^ different antibodies, consistent with theoretical analyses of library size (85). However, as library size has scaled upward (to claimed diversities of >10^11^), the number of antibodies selected against individual targets has generally remained below 100 in the absence of heroic efforts (81). The use of deep sequencing described here, and elsewhere (44, 47–50), indicates that the gap between the potential diversity of selectable antibodies, and the significantly lower number usually analyzed is predominantly a sampling problem, which can be overcome with ongoing improvements in sequencing technology. This will allow the calculation, rather than the estimation, of the true diversity of antibody repertoires and antigen-specific populations selected from them.

## Materials and Methods

### Bacterial and Yeast Strains

DH5aF′: F′/endA1 hsdR17(rKmK+) supE44 thi-1 recA1 gyrA (Na1r) relA1 D(lacZYAargF) U169 (m80lacZDM15)
Omnimax (Life Technologies): F′ {proAB lacIq lacZM15 Tn10(TetR) (ccdAB)} mcrA (mrr hsdRMS-mcrBC) 80(lacZ)M15 (lacZYAargF)U169 endA1 recA1 supE44 thi-1 gyrA96 relA1 tonA panD
EBY100 (kindly provided by Prof. Dane Wittrup): MATa AGA::GAL1-AGA1::URA3 ura3-52 trp1 leu2-delta200 his3-delta200 pep4::HIS3 prb11.6R can1 GAL

### scFv Antibody Selections

*In vivo* biotinylated His-tagged CDK2 protein (NP_001789.2), produced by the SGC (Toronto) was used for the scFv phage display selections. The naïve scFv library described in the study by Sblattero and Bradbury (59) was used for two rounds of phage display against the antigen with streptavidin magnetic beads. Two additional rounds of yeast display sorting were performed using 100 nM of antigen. The detailed protocol for antibody selections against biotinylated proteins is described in the study by Ferrara et al. (45).

For the selection of clones sharing same HCDR3 derived from the naïve library and cloned into yeast display vector (see below), cells were induced and labeled with anti-SV5-PE to assess the scFv display level.

All the flow cytometry-assisted sorting experiments were performed using the FACSAria (Becton Dickinson) sorter and analyzed using FlowJo software (FlowJo LLC).

When single clones were analyzed for their specificity, they were stained with CDK2, unrelated antigen, and conjugated streptavidin, as negative controls. All experiments with single clones were performed in a 96-well format using the LSRII (Becton Dickinson) flow cytometer.

### Next-Generation Sequencing

The plasmid DNA of the anti-CDK2 second sort output was used as a template for the PCR targeting the HCDR3 region of the scFvs. A set of forward primers mapping to the framework region upstream of the HCDR3 and carrying one of the Ion Torrent sequencing adaptors were used in combination with a barcoded reverse primer mapping to the common SV5 tag region of the yeast display vector and carrying the second adaptor required for sequencing. The primer sequences and method are described in detail by D’Angelo et al. (61). Once amplified with the proofreading Phusion polymerase (NEB), gel extracted, and quantified (Q-bit, HS-DNA kit, Invitrogen), the amplicon libraries were processed using the Ion Xpress Amplicon library protocol and then prepared for sequencing on the Ion 316 Chip (Life Technologies). The sequences analysis was performed using the AbMining Toolbox as described by D’Angelo et al. (61).

### Primer Design and Inverse PCR

The inverse PCR strategy is described in the study by D’Angelo et al. (50). Briefly, primers were designed on the DNA consensus sequence for the HCDR3 of the top-ranked clone as back to back primers directed outward from the middle of the HCDR3, with a 5′ phosphorylated forward primer. The inverse PCR was carried out using a high-fidelity polymerase with proofreading activity (Phusion High Fidelity Polymerase, NEB) and either 0.03 fmol of plasmid DNA obtained from the yeast sorted population (1,000–10,000 times the diversity of the sorting output) or 0.3 fmol of the original phage naïve library were used as a template.

After amplification, the PCR product was gel extracted and purified (Qiaquick Gel extraction kit, Qiagen) to avoid contamination from the original plasmid template. The purified products were ligated with T4 ligase and transformed into DH5aF′ bacterial cells.

For the clones obtained from the second yeast sort enriched for CDK2 binders, single clones were analyzed by Sanger sequencing to confirm the presence of the correct HCDR3 and obtain the sequence of the full-length scFv, before carrying out binding assays. The sequenced plasmid clones were then retransformed into the EBY100 yeast display strain (Yeast transformation kit, Sigma) for testing by flow cytometry.

When the entire unselected naïve library was used as a template to isolate clones sharing the same HCDR3 by inverse PCR, ligation and bacteria transformation were performed. A plasmid preparation was obtained and transformed into the EBY100 yeast display cells. The final product was a sublibrary of scFvs, with 10^6^ clones sharing the same HCDR3.

### Affinity Measurement

The affinity of the selected clones was determined by yeast display using the equilibrium binding titration curve to extrapolate the equilibrium dissociation constant (K_D_), as described by Boder and Wittrup (86). Briefly, the induced, monoclonal populations of yeast displaying scFvs were incubated with eight concentrations of biotinylated antigen, ranging from 1 to 500 nM for 30 min at room temperature to allow the binding reaction to reach equilibrium followed by 5 min on ice for 5 min to reduce the off-rate. After washing, the yeast cells were incubated with the secondary reagents (streptavidin-Alexa633, to detect antigen binding to the displayed scFvs, and anti-SV5-PE, to detect the yeast displayed scFvs) on ice for 30 min. After the final washes, the samples were analyzed by flow cytometry on the BDAriaIII (BD Biosciences). The mean fluorescence intensities of the gated binding/displaying populations were plotted against the antigen concentration, and a non-linear least-squares analysis was used to fit the curve and obtain the K_D_ values for each scFv.

### *In Vivo* Database Analysis

In the first database (63), the identity of VH, DH, and JH genes making up each individual HCDR3 were determined on the basis of the nucleotide sequences using IMGT (87) and NCBI IgBlast software (88), along with a CDR3 motif identification algorithm (89). The final data set comprising HCDR3 sequences found in three individuals was kindly provided by DeKosky and Georgiou. The second database (64) used a scored alignment across a definition list of all the known VH, DH, and JH genes in IMGT (90). The naïve data sets for three individual donors were downloaded from the public repository found at http://adaptivebiotech.com/pub/robins-bcell-2016.^1^ Data were filtered through RStudio software package [RStudio Team (2015) RStudio: Integrated Development for R. RStudio, Inc. (Boston, MA, USA)],^2^ whereby 7.4 × 10^6^, 6.0 × 10^6^, and 8.4 × 10^6^ individual sequences were processed for the three separate donors, respectively. In some cases, the families could be identified, but not the individual germline VH, DH, or JH genes. Therefore, this initial data set was processed further to include only complete data sets (e.g., HCDR3, VH, DH, JH designations). Any sequence that contained multiple gene or family designations and/or stop codons within HCDR3 was excluded. The final curated set consisted of 2.6 × 10^6^, 2.4 × 10^6^, and 3.6 × 10^6^ in the three respective donors, of which the unique HCDR3 sequences and VH, DH, and JH gene recombination were tabulated.

## Author Contributions

SD and FF equally contributed to this work. SD, FF, and AB contributed to research design. SD, FF, and LN conducted experiments; SD, FF, PH, and ME performed data analysis. SD, FF, ME, PH, and AB wrote the manuscript.

## Conflict of Interest Statement

SD, FF, LN, FE, and AB are employees of Specifica Inc. The remaining author declares that the research was conducted in the absence of any commercial or financial relationships that could be construed as a potential conflict of interest.

## Footnotes

^1^http://doi.org/10.21417/B71018.

^2^http://www.rstudio.com/.
